# Proteome–Transcriptome Discordance in Rice Under Drought Is Modulated by Post-Translational Modifications with Functional Consequences for Photosynthesis and Energy Metabolism

**DOI:** 10.3390/plants15101559

**Published:** 2026-05-20

**Authors:** Zhiyu Guo, Xiaohao Yan, Jiansheng Liang

**Affiliations:** 1 Shenzhen Key Laboratory of Plant Genetic Engineering and Molecular Design, Institute of Plant and Food Science, Southern University of Science and Technology, Shenzhen 518055, China; 12333170@mail.sustech.edu.cn (Z.G.); 12231421@mail.sustech.edu.cn (X.Y.); 2 Department of Biology, School of Life Sciences, Southern University of Science and Technology, Shenzhen 518055, China

**Keywords:** proteome, transcription, post-translation modification, drought stress, photosynthesis, energy metabolism

## Abstract

Transcriptome profiling has been widely used to dissect the molecular mechanisms underlying plant responses to environmental stresses, yet the extent to which RNA changes reflect functional protein levels remains unclear. Here, we performed an integrated multi-omics analysis of the transcriptome, proteome, phosphoproteome, and acetylome in rice during a drought–rewatering cycle. We first identified 5449 differentially expressed genes (DEGs) and 525 differentially expressed proteins (DEPs) under drought stress, followed by 4340 DEGs and 328 DEPs upon rewatering, which underpinned an extensive remodeling of photosynthetic and metabolic pathways. Temporal clustering of transcriptomic and proteomic data then delineated five distinct expression patterns for both transcripts and proteins, uncovering transcriptional and translational strategies ranging from rapid reversal to persistent stress adaptation. Despite the observed coherence in some expression clusters, we nonetheless uncovered widespread transcriptome–proteome discordance, with a substantial fraction of gene–protein pairs exhibiting uncorrelated abundance changes. Remarkably, the observed discordance is quantitatively associated with the dynamic nature of post-translational modifications, including phosphorylation and acetylation, which act as key post-transcriptional tuners to independently regulate protein abundance—particularly for components of photosynthesis and energy metabolism—enabling plants to dynamically balance stress tolerance with the maintenance of core physiological functions. Our research delves into the intricate and often distinct regulatory networks that span transcriptional, translational, and post-translational levels, extending beyond a singular transcriptional focus.

## Simple Summary

**Significance Statement:** Our findings extend beyond a transcription-centric view by revealing complex, coordinated, yet frequently decoupled regulatory hierarchies spanning transcriptional, translational, and post-translational levels. This integrative framework elucidates the dynamic regulation mechanism that plants exert between stress tolerance and post-stress recovery, providing mechanistic insights into drought adaptation strategies and serving as a crucial resource for enhancing crop resilience.

## 1. Introduction

Rice (*Oryza sativa* L.) is the grain most widely consumed as a staple food by people worldwide, particularly in Asian countries [1]. Climate change has resulted in more frequent and severe drought stress, which has become a major abiotic stress factor, hampering rice production and stable yields [2]. While transcriptomic profiling has served as the principal methodology for dissecting plant stress responses and has facilitated the identification of numerous drought-responsive genes and signaling pathways [3,4,5], whether transcriptional changes fully represent the functional state of cells requires further validation at the proteomic level and beyond.

The classic “central dogma” states that genetic information is transferred from DNA to RNA to protein. Proteins, as the primary functional executors in cells, are regulated through multiple sophisticated layers including transcription, post-transcription, translation, and post-translation [6]. Key determinants include RNA abundance, translational efficiency governed by codon usage and mRNA structural motifs, gene length and the stability and turnover rate of the protein itself, which is extensively regulated by post-translational modifications (PTMs) such as phosphorylation and acetylation [7,8,9]. Numerous studies in human and mouse tissues have demonstrated the discordance between RNA and protein [10,11], suggesting that there are molecular mechanisms affecting protein abundance and functions. For instance, phosphorylation exerts a dual role in regulating protein abundance, where specific sites can either accelerate degradation to maintain cellular homeostasis or unexpectedly enhance protein stability [8]. Acetylation enhances protein stability through two distinct mechanisms: by modulating structural folding to promote conformational stability, and by competitively inhibiting ubiquitination to impede proteasomal degradation [12]. Meanwhile, dysregulation of PTMs is linked to metabolic disorders, highlighting the therapeutic promise of targeting these modifications in clinical practice [13,14]. In *Arabidopsis*, the regulatory mechanisms underlying nearly half of the variation in protein and RNA abundance are still uncharacterized [15]. Recent studies have demonstrated the post-transcriptional regulatory role of N^6^-methyladenosine (m^6^A) in mediating the discordance between protein and RNA abundance in rice and soybean [16,17]. However, the specific contributions of the proteomic modificome, including various types of protein modifications such as phosphorylation and acetylation, to the observed discordance in plant stress responses are not well understood.

Recent technological advancements in high-throughput sequencing and high-resolution mass spectrometry have significantly enhanced our ability to simultaneously quantify transcripts, proteins, and their post-translational modifications at a large scale in plants, shedding light on their critical roles in plant biology. Foundational work from plant research groups has been instrumental in establishing the methodological and conceptual framework for plant proteomics and PTM profiling. Previous studies established the basis for quantitative plant proteomics, subcellular proteome atlases, and multi-omics integration long before such approaches became widespread, providing indispensable resources and insights that underpin subsequent studies [18,19,20]. A landmark study employing a TMT-based proteomic strategy quantified over 15,000 rice proteins, establishing an invaluable resource and underscoring the necessity of direct proteomic measurement to complement transcriptomic data [16]. The recently established GreenPhos platform substantially advances plant phosphoproteomics by enabling efficient phosphopeptide purification from diverse species including *Arabidopsis*, rice, and tomato, achieving unprecedented coverage of approximately 11,000 phosphosites through streamlined single LC-MS runs [21]. A breakthrough in plant acetylome research established an extensive resource of 7456 acetylation sites on 2638 proteins, nearly two-thirds of which were novel identifications, greatly expanding the known landscape of this modification in plants [22]. PTM-based proteomics has been extensively applied to investigate dynamic responses to abiotic stresses in plants. Phosphoproteomic studies in species including *Arabidopsis*, rice, and maize have elucidated the central role of kinase networks in drought signal transduction [22,23,24]. Meanwhile, acetylome investigations have demonstrated that numerous enzymes involved in drought stress responses—particularly those governing photosynthesis and energy metabolism—undergo extensive acetylation, thereby modulating their activity [25,26,27]. However, the coordinated modulation of protein activity by multiple PTM layers and their contribution to the discordance between transcriptomic and proteomic profiles during drought stress are not well understood.

In this work, we conducted an integrated multi-omics study to systematically profile the transcriptome, proteome, phosphoproteome, and acetylome in rice during drought stress and rewatering (post-drought recovery). Our findings demonstrate a widespread transcriptome–proteome discordance in rice, with genes showing such discordance being significantly enriched in photosynthesis and energy metabolism pathways under drought stress. Notably, phosphorylation and acetylation modifications are specifically linked to the discordance in these functional categories, thereby mediating drought adaptation through coordinated regulation of photosynthetic performance and energy homeostasis. This study offers a refined perspective on the role of PTM-based regulation in plant drought adaptation, emphasizing its functional specificity in core physiological processes.

## 2. Results

### 2.1. Differential Expression Patterns of Genes and Proteins During Drought and Rewatering Cycle

**Figure 1:**
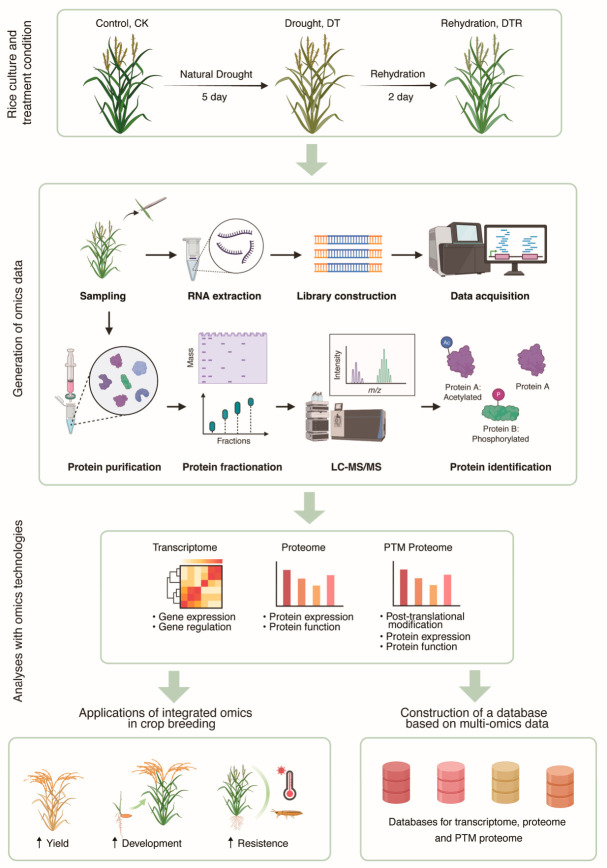
Experimental design and multi-omics workflow during drought–rewatering cycle. During the heading stage, rice plants underwent five days of natural drought treatment (DT) followed by two days of rewatering (DTR), with control plants (CK) maintained under well-watered conditions. Apical segments of 4–5 leaves (2–4 g) were collected from each treatment group and used for transcriptomic, proteomic, and PTM proteomic analyses. This figure was created with BioRender (Yan, X. (2026) https://BioRender.com/wjmecl7).

In this study, we conducted the RNA-seq and Pro-DIA analyses at the rice heading stage, a growth phase highly sensitive to drought stress and essential for yield determination. The study focuses on the accumulated molecular changes across three biologically distinct states, control (CK), established drought stress (DT), and post-rewatering recovery (DTR), capturing the cumulative regulatory landscape before and after drought stress and recovery. Drought treatment (DT) and rewatering post-drought stress (DTR) were applied to assess transcriptomic and proteomic alternations (Figure 1) and to elucidate the molecular mechanisms underlying drought responses associated with differentially expressed genes and proteins.

**Figure 2:**
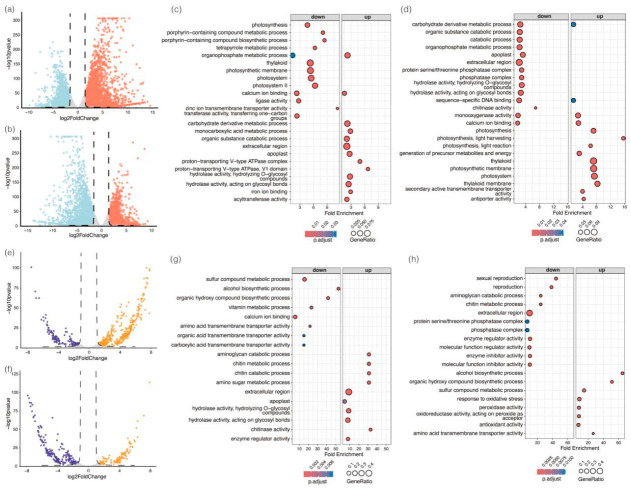
Differential expression analysis under drought stress and rewatering. (**a**,**b**) Volcano plots showing differentially expressed genes (DEGs) in DT vs. CK and DTR vs. DT (**b**) based on a *q*-value < 0.01 and |log2FoldChange| > 1.5. (**c**,**d**) GO enrichment analysis of up-regulated and down-regulated genes in DT vs. CK (**c**) and DTR vs. DT (**d**) ranked by *p*-value. (**e**,**f**) Volcano plots showing differentially expressed proteins (DEPs) in DT vs. CK (**e**) and DTR vs. DT (**f**) based on a *p*-value < 0.05 and |log2FoldChange| > 1.2. (**g**,**h**) GO enrichment analysis of up-regulated and down-regulated proteins in DT vs. CK (**g**) and DTR vs. DT (**h**) ranked by *p*-value.

To ensure the reliability of the transcriptomic dataset, RNA-seq libraries generated 19,023,434 to 23,575,622 clean reads across samples, with an average alignment rate of 98.86% and a high reproducibility among biological replicates (Pearson’s r = 0.97–1) (Figure S1a,b). In the DT vs. CK (control treatment) comparison, a total of 5449 differentially expressed genes (DEGs) in the drought-treated plants compared to the control were identified, with 3180 genes up-regulated and 2269 genes down-regulated, based on a q-value < 0.01 and |log_2_FoldChange| > 1.5 (Figure 2a). Down-regulated genes were primarily enriched in photosynthesis-related pathways, including photosystem complexes and porphyrin metabolism, suggesting that these genes are responsible for the repression of photosynthetic capacity during drought (Figure 2c). Conversely, the up-regulated genes were associated with carbohydrate metabolism, monocarboxylic acid processes, and hydrolase activity, implying metabolic reprogramming to support resource redistribution under stress. In the DTR vs. DT comparison, a total of 4340 DEGs were identified (*q*-value < 0.01 and |log_2_FoldChange| > 1.5), with 1757 genes mainly responsible for photosynthesis-related processes up-regulated, whereas 2583 genes associated with the catabolic pathways were down-regulated (Figure 2b,d), highlighting a rapid recovery of energy metabolism after rewatering. The transcriptomic signatures imply that metabolic reprogramming is reversed within plant cells during drought and rewatering cycles, regulating the allocation of resources and energy between drought tolerance and growth.

To characterize the responses of rice plants to drought and rewatering at the protein level, we quantified the dynamics of protein abundance using label-free liquid chromatography tandem mass spectrometry (LC-MS/MS) during the drought and rewatering treatment cycle. We identified 7130 proteins and 44,787 peptide fragments, with 6616 to 6987 proteins quantified for different treatments (Figure S2a,b). The Pearson’s correlation and principal component analysis (PCA) revealed a clear separation among treatments and strong clustering within replicates (Figure S2c,d), establishing a robust foundation for further analysis. A total of 525 differentially expressed proteins (DEPs) were identified in the DT vs. CK comparison (|log_2_ FoldChange| > 1.2 with a *p*-value < 0.05), including 371 up-regulated and 154 down-regulated proteins, respectively (Figure 2e). GO enrichment analysis showed that drought-repressed proteins were mainly involved in organic compound biosynthesis and transmembrane transport, whereas drought-induced proteins were enriched in chitin and amino sugar metabolism and in hydrolase activities (Figure 2g). Upon rewatering, a total of 328 DEPs were identified (|log_2_ FoldChange| > 1.2 with a *p*-value < 0.05) in the DTR vs. DT comparison, including 113 up-regulated proteins enriched in the oxidative stress response and antioxidant activity, and 215 down-regulated proteins involved in catabolic processes and enzyme activity regulators, respectively (Figure 2f,h). These results imply a significant metabolic remodeling occurred during drought and subsequent rewatering in rice.

### 2.2. Temporal Transcriptomic Clustering Reveals Distinct Drought Response Strategies

**Figure 3:**
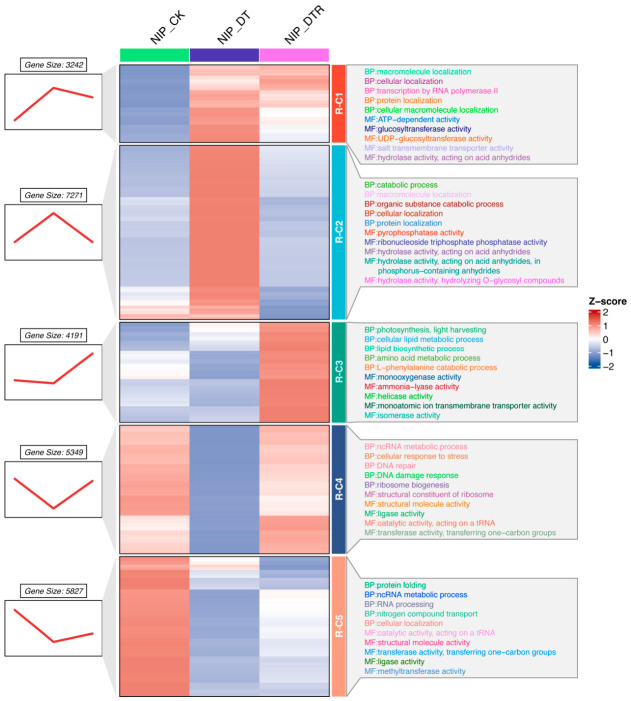
Identification of specific clusters of transcripts. Five gene clusters (R-C1 to R-C5) were identified based on their distinct temporal expression patterns using Mfuzz clustering. For each cluster, the top five enriched Gene Ontology (GO) biological processes and molecular functions were identified and ranked by *p*-value.

The expression levels of plant genes are profoundly influenced by environmental fluctuations and respond dynamically to external stimuli, thereby modulating key biological processes, particularly those associated with stress adaptation. To characterize these transcriptional dynamics during drought stress and subsequent rewatering, a time-series analysis was performed and five distinct expressional clusters with unique temporal trajectories and functional signatures were identified (Figure 3).

RNA-cluster 1 (R-C1), consisting of 3242 genes, exhibited increased expression during drought followed by only partial reversal after rewatering. Functional enrichment analysis indicated that these genes contribute to cellular organization and transcriptional regulation. Their incomplete recovery suggests the presence of an adaptive transcriptional memory. The genes clustered in R-C3 (with 4191 genes) maintained stable expression during drought but showed marked increase in expression after rewatering. These genes were enriched in processes related to photosynthesis, lipid metabolism and amino acid metabolism, suggesting their specialized functions in re-establishing energy production and metabolic processes during recovery. The genes in R-C5, including 5827 genes, showed decreased expression during drought and only partial recovery after rewatering. These genes are involved in protein folding and RNA processing, implying persistent impairment of cellular maintenance systems.

The characteristics of gene expression patterns in R-C2 (comprising 7271 genes) exhibited up-regulation in response to drought and fully recovered following rewatering. These genes are predominantly involved in catabolic pathways, representing reversible metabolic responses that enable rapid restoration of homeostasis. In contrast, R-C4 genes (encompassing 5349 genes) exhibited decreased expression under drought and nearly complete recovery upon rewatering. Functional enrichment analysis indicated that these genes are involved in DNA repair and ribosome biogenesis, supporting the restoration of genomic integrity and protein synthesis machinery after stress release.

Collectively, these five distinct expression patterns illustrate the intricate transcriptional reprogramming that occurs during the drought–rewatering cycle in rice. The co-existence of completely reversible (R-C2, R-C4) and partially reversible (R-C1, R-C5) patterns suggests that rice simultaneously employs transient responses for immediate stress relief and persistent regulatory mechanisms for long-term adaptation. Moreover, the delayed activation observed in R-C3 reflects a distinct mechanism dedicated to metabolic restoration during recovery. These insights underscore the temporal complexity of drought-responsive gene regulation and reveal coordinated regulatory programs that orchestrate stress resilience and post-stress recovery.

### 2.3. Proteome-Level Strategies Underlying Drought Stress Adaptation

**Figure 4:**
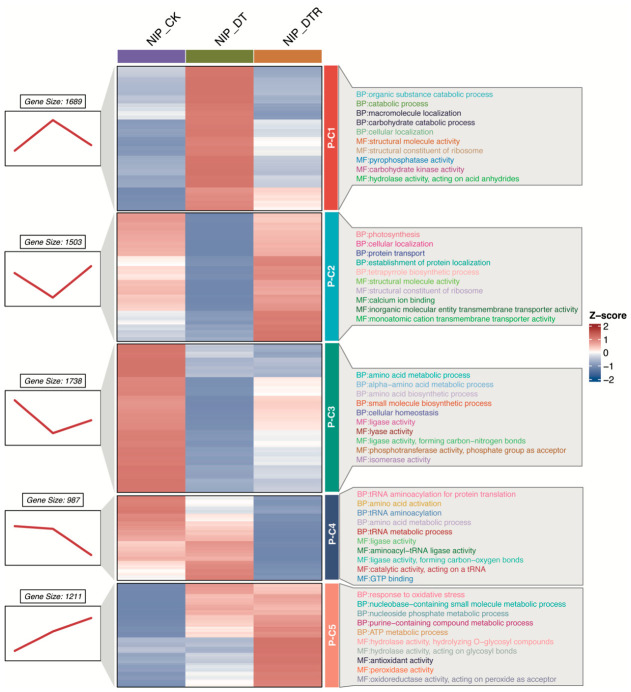
Dynamic protein expression patterns under drought stress. Five protein clusters (P-C1 to P-C5) were identified based on their distinct temporal expression patterns using Mfuzz clustering. For each cluster, the top five enriched Gene Ontology (GO) biological processes and molecular functions were identified and ranked by *p*-value.

To elucidate the protein-level regulatory landscape underlying their responses to drought and subsequent recovery, we characterized five distinct protein expression clusters based on their temporal dynamics during drought and rewatering, each reflecting a unique regulatory strategy, similar to the transcriptomic analysis (Figure 4).

The protein expression of protein cluster (P-C1, including 1689 proteins) showed significant stimulation under drought and fully returned to the pre-treatment levels upon rewatering. These proteins were enriched in organic substance catabolic processes, suggesting their essential roles in metabolic adjustment during the drought–rewatering cycle. In contrast, the expression of proteins in P-C2 (with 1503 proteins) was significantly down-regulated during drought but substantially increased after rewatering. Functional enrichment showed strong associations with photosynthesis and tetrapyrrole biosynthesis, highlighting both the vulnerability and the resilience of photosynthetic machinery under drought stress.

The amino acid metabolism and cellular homeostasis-related proteins in P-C3, consisting of 1738 genes, exhibited drought-repressed expression with only partial recovery upon rewatering, suggesting a sustained metabolic alteration and reprogramming. The proteins clustered in P-C4 (comprising 987 proteins) maintained stable abundance during drought but displayed a marked decline after rewatering, implying a stress-induced energy conservation strategy followed by resource reallocation during recovery. Finally, the expression of the P-C5 clustered proteins (encompassing 1211 proteins) exhibited a continuous increase throughout both drought and rewatering periods. Enrichment analyses indicated the involvement of purine metabolism and antioxidant activity, reflecting a progressive reinforcement of oxidative stress defense mechanisms.

Taken together, these distinct temporal expression patterns at proteomic levels reveal that rice employs a multi-layered proteomic strategy to cope with drought stress, integrating reversible responses, persistent metabolic shifts, and delayed regulatory programs to enable both survival during drought and efficient recovery thereafter.

### 2.4. Synchronized Expression Dynamics Between Transcripts and Proteins During Drought and Rewatering

The distinct expression patterns observed at both transcriptomic and proteomic levels during the drought–rewatering cycle suggest that rice plants deploy a cost-effective, rapid, and highly adaptable response to drought stress. To obtain an integrated view of stress tolerance, we performed a joint analysis of transcriptional and translational dynamics. We first compared the number of genes detected at the RNA level with their corresponding proteins and quantified the proteins encoded by transcripts with log_2_(TPM) > 1. Notably, a majority of the transcripts lacking protein evidence displayed high RNA abundance, whereas genes detected at both RNA and protein levels were mainly distributed within the low- to medium-expression range across samples (log_2_(TPM) < 5, Figure S3a–c). This expression pattern suggests substantial filtering between transcription and translation, and many highly expressed transcripts tended to be translated. Moreover, these distribution patterns showed minimal variation among treatments, suggesting that the degree of transcript–protein filtering remains largely stable under drought and rewatering conditions.

**Figure 5:**
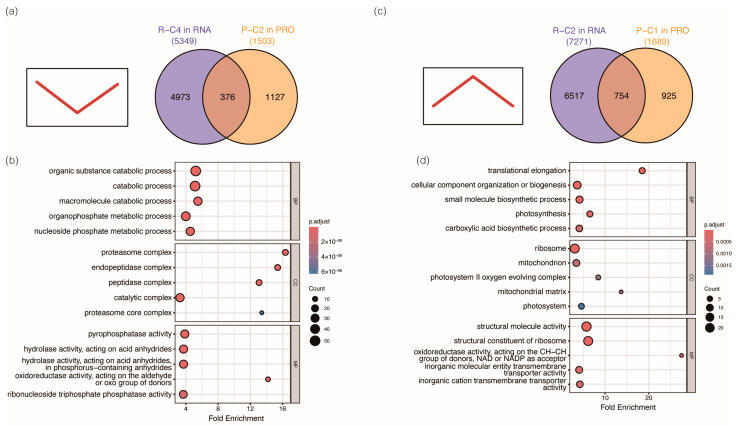
Consistent dynamic expression patterns across transcriptional and translational layers. (**a**) The Venn diagram shows the overlap of genes whose RNA and protein expression were both down-regulated under drought and exhibited complete recovery after rewatering, corresponding to the R-C4/P-C2 cluster pair. (**b**) GO enrichment analysis of the overlapping genes/proteins in the R-C4/P-C2 module. (**c**) The Venn diagram shows the overlap of genes whose RNA and protein expression were both up-regulated during drought followed by complete normalization, corresponding to the R-C2/P-C1 cluster pair. (**d**) GO enrichment analysis of the overlapping genes/proteins in the R-C2/P-C1 module.

We next focused on the cases where transcripts and protein abundance exhibited consistent expression patterns across the drought–rewatering cycle. In the R-C4/P-C2 cluster pair, which showed down-regulated expressions of both genes and proteins under drought and fully recovered after rewatering, there were 5349 genes and 1503 proteins exhibiting the same down-regulated expression pattern when exposed to drought treatment; only 376 genes and their coding proteins were identified at the same time in this cluster pair. The functions of these shared genes and proteins were mainly responsible for organic substance catabolic processes and protein complexes (Figure 5a,b). In contrast, in the R-C2/P-C1 cluster pair, where the expression patterns at both the transcriptional and translational levels were up-regulated during drought and recovered to the pre-treatment levels after rewatering, 754 genes and their encoded proteins were identified as sharing the same expression patterns, which represents 10.37% of genes and 44.64% proteins, respectively (Figure 5c). These genes/proteins were functionally specialized in cellular component biosynthetic processes and photosynthetic processes (Figure 5d). In the third expression pattern, like the R-C5/P-C3 cluster pair, where the expression levels of both genes and proteins were down-regulated after drought treatment and only partially recovered post-rewatering, there were 567 genes and their encoded proteins, showing a similar expression pattern, which represented 9.73% for genes and 32.62% for proteins, respectively, and these genes/proteins were functionally linked to amino acid metabolism, protein folding and cellular homeostatic processes (Figure S3d). These results indicate that the journey from a gene to a functional protein is governed by multiple regulatory checkpoints. The observed consistency and discordance between the transcriptome and proteome during drought stress and rewatering highlight that the Central Dogma (DNA → RNA → Protein) is a simplified roadmap, not a complete picture of the intricate regulatory landscape within a cell. The responses of plants to drought stress involve complex coordination across transcriptional, translational, and post-translational processes, reflecting trade-offs between rapid response, resource allocation, and sustained reprogramming of cellular homeostasis when exposed to the fluctuating environmental stresses.

### 2.5. Discordance Between Transcripts and Proteins Under Drought and Rewatering

The pronounced disparities between transcriptional and proteomic responses to drought stress prompted us to quantitatively assess the extent and functional implications of regulatory divergence across molecular layers. We first employed the protein-to-RNA ratio (PTR) as an indicator of translational coordination [15]. Although the overall PTR distributions showed substantial overlap among treatments with no statistically significant differences (Kruskal–Wallis test, *p* = 0.74) (Figure S4a), drought stress caused a modest reduction in PTR values, followed by partial recovery upon rewatering (Figure S4b).

**Figure 6:**
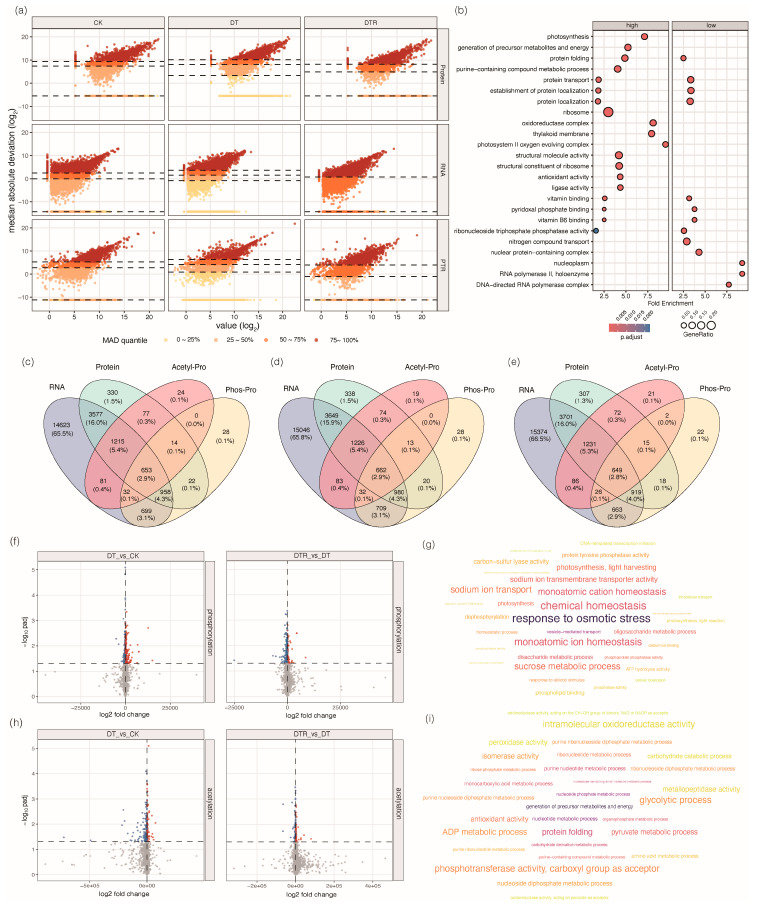
Assessment of transcriptome–proteome discordance. (**a**) Expression variability analysis using the median absolute deviation (MAD) for proteins, RNA, and protein-to-RNA ratios (PTR) across different treatment conditions. For each gene/protein, RNA abundance was quantified as log_2_(TPM + 1), and protein abundance was quantified based on Pro-DIA intensity. PTR was calculated as the difference between protein and RNA abundance (PTR = log_2_(protein abundance) − log_2_(TPM + 1)). Variability within each treatment group was assessed by computing MAD, defined as the median of the absolute deviations from the median (MAD = median(|Xi − median(X)|)). (**b**) GO enrichment analysis of high-PTR and low-PTR genes under drought treatment. (**c**–**e**) Venn diagrams showing the overlap of genes and their encoding proteins identified across transcriptomic, proteomic, phosphoproteomic, and acetylomic datasets in CK (**c**), DT (**d**), and DTR (**e**) treatments. (**f**) Volcano plot of differentially phosphorylated sites in DT vs. CK (**left**) and DTR vs. DT (**right**). The red dots represent upregulated phosphorylated sites, blue dots represent downregulated phosphorylated sites, and gray dots represent non-significant changes. (**g**) Gene Ontology (GO) enrichment analysis of genes associated with differentially phosphorylated sites. (**h**) Volcano plot of differentially acetylated sites in DT vs. CK (**left**) and DTR vs. DT (**right**). The red dots represent upregulated acetylated sites, blue dots represent downregulated acetylated sites, and gray dots represent non-significant changes. (**i**) Gene Ontology (GO) enrichment analysis of genes associated with differentially acetylated sites.

To further capture regulatory variability across treatments, we applied the median absolute deviation (MAD) analysis to transcripts, proteins, and PTR [16]. MAD reflects the degrees of variation or dispersion of transcripts, proteins and PTR, and therefore, serves as an indicator of stress-induced regulatory activation. Drought stress disrupts transcriptional–translational coordination and enhances their decoupling (Figure 6a). In the control group, the transcripts, proteins and PTR exhibited balanced MAD distributions, reflecting well-coordinated transcriptional–translational coupling. Drought treatment caused upward shift of the MAD of both transcripts and proteins, suggesting large-scale activation of stress-responsive pathways. Concurrently, PTR values exhibited more dispersed patterns under drought stress, reflecting an enhanced transcription–translation decoupling, which was suggested to be likely driven by changes in translational regulation, protein turnover, or post-translational processes. After rewatering, the MAD of transcripts and proteins partially returned, suggesting a prolonged adjustment. The variation in PTR decreased but did not return to pre-stress levels after rewatering, indicating a persistent dysregulation between transcripts and protein synthesis.

To elucidate the functional bases of these differences in translation efficiency, we performed GO enrichment analysis on genes stratified by PTR. Under drought and rewatering, high-PTR genes (median + 2 × MAD) were enriched in ribosomal components, protein folding, and photosynthetic system, while low-PTR genes (lowest 25%) were associated with transcriptional regulation and metabolic adjustment (Figure 6b and Figure S4d). Notably, these functional enrichments were distinct from those observed under the control condition (Figure S4c), suggesting that genes with different PTR values adopt environment-specific expression programs in rice.

To investigate the potential regulatory mechanisms underlying transcript–protein discordance, we conducted comprehensive phosphor-proteomic and acetylomic profiling. The phosphoproteome comprised 6755 phosphopeptides corresponding to 2658 proteins and 6976 sites, with 5140, 5178, and 4613 sites detected in CK, DT, and DTR, respectively (Figure S5a–c). The acetylome included 5679 acetylated peptides mapped to 2112 proteins and 5624 sites, with a comparable number of sites detected across treatments (Figure S6a–c). Both datasets showed high reproducibility (Pearson’s r = 0.97–1), confirming robustness for further analyses (Figures S5d,e and S6d,e). Cross-layer quantification revealed that genes with transcript evidence far exceeded those with detectable protein or PTM evidence (Figure 6c–e), and the transcription factors (TFs) displayed distinct regulatory behaviors across RNA, protein, and PTM layers (Figure S7a–c), suggesting extensive multi-layer regulation during stress adaptation. Under drought treatment, the classical stress-responsive TF families, including MYB, HSF, and ERF, exhibited marked increases at both the protein and PTM levels, especially in phosphorylation, suggesting activation beyond transcriptional induction. In contrast, TFs, such as bHLH and ARF, showed little changes at the protein level but reduced PTM levels. After rewatering, several TF families (e.g., bZIP, bHLH, C2H2) maintained high abundance of both protein and PTM, whereas typical stress-related TFs, such as HSF, WRKY, and MYB, declined sharply, reflecting selective regulatory requirements during recovery.

We next characterized the drought-responsive phosphorylation and acetylation sites. Most phosphoproteins carried only a single phosphorylation site (46.3%), whereas proteins with two phosphorylation sites accounted for ~21% (Figure S5f). The phosphorylation predominantly occurred on serine residues (~80%), followed by threonine residues (~15%) and tyrosine residues (Figure S5g). Similarly, 46% of acetylated proteins harbored a single acetylation site, and ~21.8% of proteins contained two acetylation sites (Figure S6f,g). The acetylation exclusively occurred on lysine residues. Functional analysis further indicated that proteins harboring differentially phosphorylated sites were enriched in osmotic stress responses, chemical homeostasis, and photosynthetic processes (Figure 6f,g), whereas those with altered acetylation were mainly associated with the precursor metabolite generation, organic substance metabolism, and phosphotransferase activities (Figure 6h,i). Taken together, these results imply that protein phosphorylation and acetylation constitute key regulatory nodes that reprogram cellular functions beyond transcriptional control to facilitate adaptive responses to drought and recovery.

### 2.6. PTM-Driven Modulation of Transcript–Protein Regulatory Divergence

**Figure 7:**
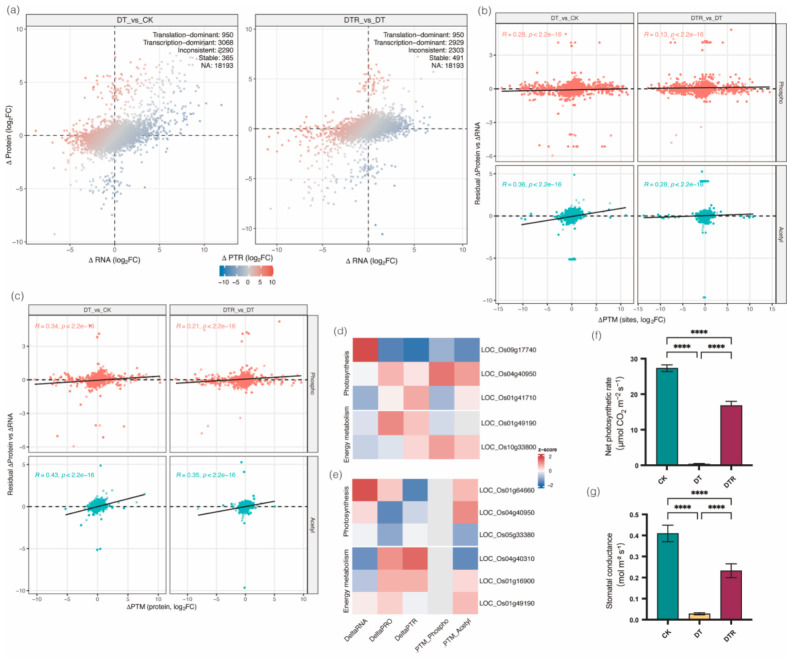
Phosphoproteome and acetylome dynamics associated with transcript–protein discordance. (**a**) Classification of gene–protein pairs by relative RNA and protein changes. Gene–protein pairs were classified into four categories according to the direction and relative magnitude of changes in RNA and protein abundance in the two contrasts (DT vs. CK and DTR vs. DT). Fold changes for RNA and protein were calculated as log_2_ fold change (log_2_FC); differential expression thresholds were set as follows: RNA, |log_2_FC| > 1.5 and adjusted *p* < 0.01; protein, |log_2_FC| > 1.2 and *p* < 0.05. For each pair, categories were assigned by comparing ΔRNA and ΔProtein: Translation-dominant was defined as |ΔProtein| ≥ 2 × |ΔRNA| and |ΔProtein| exceeds protein significance threshold (i.e., protein change dominates over RNA change). Transcription-dominant was defined as |ΔRNA| ≥ 2 × |ΔProtein| and |ΔRNA| exceeds RNA significance threshold. Inconsistent was defined as ΔRNA and ΔProtein have opposite signs (one up, the other down) and at least one meets the significance threshold. Stable was neither ΔRNA nor ΔProtein meet the significance thresholds or both change in the same direction but below dominance criteria. “NA” indicates no evident change. (**b**) Site-level correlation between PTM changes and residual ΔProtein. To quantify the contribution of post-translational modifications (PTMs) to transcript–protein discordance, we first computed the residual ΔProtein for each gene within each contrast. For each treatment contrast, a linear model, ΔProtein = β0 + β1 × ΔRNA + ε, was fitted using all quantified gene–protein pairs, and the residual term (residual ΔProtein vs. ΔRNA) was extracted to represent the deviation of the observed protein change from that predicted by its RNA change. PTM site-level changes (ΔPTM_site) were defined as the contrast-specific log_2_FC of PTM intensity for each phosphorylation or acetylation site. Residual protein changes were then mapped back to PTM sites through their corresponding genes. Correlation analysis was performed at the site level by relating ΔPTM_site to residual ΔProtein vs. ΔRNA using Spearman’s rank correlation. Scatter plots show individual PTM sites with linear regression fits, faceted by PTM type (phosphorylation or acetylation) and treatment contrast. Spearman correlation coefficients (R) and two-sided *p*-values are displayed within each panel. (**c**) Protein-level correlation between aggregated PTM changes and residual ΔProtein. Complementing the site-level analysis, we next aggregated PTM information to the protein level. For each protein and contrast, all PTM sites belonging to the same modification type (phosphorylation or acetylation) were summarized using the median site-level ΔPTM value (dPTM_pro), reflecting the overall direction and magnitude of protein-level PTM regulation. Residual protein changes (residual ΔProtein vs. ΔRNA), derived from contrast-specific linear models of ΔProtein versus ΔRNA, were then mapped to the aggregated PTM values. Spearman rank correlations were computed between dPTM_pro and residual ΔProtein vs. ΔRNA to quantify the association between PTM abundance shifts and protein changes unexplained by RNA variation. Scatter plots display individual proteins with linear regression fits, faceted by PTM type and treatment contrast. Spearman correlation coefficients (R) and two-sided *p*-values are reported in each panel. (**d**,**e**) Heatmaps showing PTM-mediated regulatory strategies for selected photosynthesis and energy metabolism genes during drought stress (**d**) and rewatering (**e**). Gray boxes indicate that no corresponding post-translational sites were detected for these genes in the dataset. (**f**) Net photosynthetic rate of the top three leaves during drought–rewatering cycle (data are presented as mean ± SEM, *n* = 3–4). Significant differences compared with the control were determined by one-way ANOVA with Tukey’s test (**** *p* < 0.0001). (**g**) Stomatal conductance of the top three leaves during the drought–rewatering cycle. (Data are presented as mean ± SEM, *n* = 3–4.) **** *p* < 0.0001 (one-way ANOVA with Tukey’s test).

To systematically evaluate transcript–protein expression relationships, we classified genes into four categories based on the relative magnitudes and directions of RNA (ΔRNA) and protein (ΔProtein) changes (Figure 7a). In the DT vs. CK comparison, we identified 950 translation-dominant genes (ΔProtein >> ΔRNA), 3068 transcription-dominant genes (ΔRNA >> ΔProtein), and 2290 genes with the inconsistent gene/protein pairs (ΔProtein and ΔRNA of opposite sign). Similar results were observed in the DTR vs. DT comparison, with 950 translation-dominant genes, 2929 transcription-dominant genes, and 2303 genes with discordant pairs (Figure 7a). Notably, ~42% of all gene/protein pairs were inconsistent, underscoring the widespread roles of post-transcriptional regulation during drought adaptation.

To quantify the contribution of PTMs to this discordance, we assessed the correlation between PTM changes (ΔPTM) and the protein residual (ΔProtein unexplained by ΔRNA). Both phosphorylation and acetylation exhibited significant positive correlations with residual ΔProtein at the site levels. In the DT vs. CK comparison, phosphorylation showed a correlation coefficient of *R* = 0.28, whereas acetylation showed a stronger association with *R* = 0.36. In the DTR vs. DT comparison, the correlations remained positive, with *R* = 0.13 for phosphorylation and *R* = 0.28 for acetylation. All correlations were highly significant (*p*-value < 2.2 × 10^−16^), implying that site-specific PTM changes are consistently associated with deviations in protein abundance that cannot be explained by RNA-level changes alone (Figure 7b). These trends became even stronger when PTM measurements were aggregated at the protein level. In the DT vs. CK comparison, phosphorylated proteins showed a correlation coefficient of *R* = 0.34, whereas acetylated proteins exhibited an even higher correlation of *R* = 0.43. Similarly, in the DTR vs. DT comparison, phosphorylation and acetylation displayed correlation coefficients of *R* = 0.21 and *R* = 0.35, respectively. All correlations were highly significant (*p*-value < 2.2 × 10^−16^) (Figure 7c). These results provide strong quantitative evidence that these PTMs are major determinants of protein abundance independent of transcription.

To reveal the functional significance of PTM-mediated regulation, we examined the representative genes in key drought-responsive pathways, including osmotic adjustment, ABA signaling, photosynthesis, and energy metabolism. Strikingly, all discordant gene–protein pairs identified were associated with photosynthetic or energy metabolism-related functions (Figure 7d,e). Some genes displayed transcriptional activation accompanied by PTM-mediated suppression of protein accumulation (e.g., LOC_Os09g17740), thereby preventing the translation of transcriptional signals into protein products. Conversely, others maintained stable RNA levels yet achieved increased protein abundance through enhanced post-transcriptional or PTM-driven regulation (e.g., LOC_Os04g40950). Additional strategies were also observed, including preservation of protein levels despite transcriptional repression (LOC_Os01g49190) and PTM-enhanced stabilization leading to elevated protein-to-RNA ratios (LOC_Os10g33800). These molecular regulatory modes matched the observed physiological results (Figure 7f,g). The net photosynthetic rate dropped from 27.33 to 0.34 μmol CO_2_·m^−2^·s^−1^ under drought, reflecting widespread molecular pausing, and partially recovered to 16.81 μmol CO_2_·m^−2^·s^−1^ (61.5% of CK) after rewatering. Stomatal conductance, which reflects the degree of stomatal opening and is closely associated with photosynthesis, respiration, and transpiration, decreased dramatically from 0.4097 mol·m^−2^·s^−1^ in the control group to 0.0282 mol·m^−2^·s^−1^ under drought, representing a 93% reduction, indicating severe stomatal closure and suppressed gas exchange. Upon rewatering, stomatal conductance partially recovered to 0.2325 mol·m^−2^·s^−1^, still below the control level. Leaf water potential, an indicator of leaf water status, averaged –1.22 MPa in the control group, dropped to –2.76 MPa after drought stress, and recovered to –1.53 MPa after rewatering, confirming that drought caused substantial leaf water loss, which was restored upon re-irrigation (Figure S8). These physiological changes are consistent with the coexistence of rapid and delayed recovery mechanisms.

In summary, these results demonstrate that phosphorylation and acetylation modifications serve as key regulatory mechanisms shaping transcript–protein divergence during drought stress. By fine-tuning protein abundance in photosynthetic and energy metabolism pathways, these PTMs enable rice plants to balance stress tolerance with the maintenance and restoration of essential physiological functions.

## 3. Discussion

Compared to single-omics data analysis, the comprehensive analysis of multi-omics data is more conducive to elucidating important biological processes in plants and their regulatory networks. In the present study, we integrated the transcriptomic, proteomic, and PTM proteomic (such as phosphoproteomic and acetylomic) data and systematically delineated the multi-layered molecular regulatory network in rice plants during drought stress and subsequent rewatering.

Temporal dynamics of gene expression provide critical insights into the adaptive strategies underlying drought response and rewatering. Time-series clustering of both the transcriptome and proteome in rice not only reveals distinct expression patterns but also identifies functionally coherent biological processes that are sequentially activated or suppressed. For instance, the rapid up-regulation of cluster R-C2, enriched in hydrolase and pyrophosphatase activity, fulfills the immediate demand for osmotic adjustment and energy mobilization through enhanced hydrolysis. Previous studies have shown that overexpression of the vacuolar H^+^-pyrophosphatase (H^+^-PPase) enhanced drought tolerance in both Arabidopsis and tomato, by energizing vacuolar solute accumulation [28,29]. The sustained drought-induced down-regulation and incomplete recovery of cluster R-C5—enriched in protein folding and RNA processing—establishes a transcriptional memory that persists beyond rewatering. This enduring suppression may prime cells for subsequent stress by maintaining a repressed state of energy-intensive maintenance systems, a strategy aligned with the primed state described in stress memory studies [30,31]. Importantly, our bulk leaf analysis likely represents an averaged signal across multiple cell types, and the observed incomplete recovery may mask substantial cell-type-specific heterogeneity in stress responses. Recent advances in single-cell and spatial transcriptomics have revealed that different cell types (e.g., mesophyll, vascular, and guard cells) exhibit distinct and sometimes opposing transcriptional programs under drought and recovery conditions [32]. Therefore, the “partial recovery” observed in cluster R-C5 could reflect a composite of fully recovered cells in some lineages and persistently suppressed cells in others. Future single-cell multi-omics studies will be essential to deconvolve such cellular heterogeneity and to precisely map cell-type-specific contributions to transcriptional memory and stress adaptation. It is worth noting that while transcripts and PTMs can change on an hourly timescale, the sampling points employed here were designed to capture the cumulative, steady-state regulatory landscape associated with agriculturally relevant drought injury and post-stress recovery. Higher-resolution temporal sampling to dissect rapid and transient molecular responses at early time points will be a valuable direction for future investigation.

At the protein level, the synchronization of specific clusters with their transcriptional counterparts underscores the robustness of core stress responses. The coordinated up-regulation of the R-C2/P-C1 pair, which governs organic substance catabolism, indicates a non-negotiable demand for alternative energy and resource reallocation. This pattern reflects a fundamental plant strategy under environmental stresses, redirecting limited resources from meeting normal growth requirements to bolstering resilience against adverse environmental conditions, which is tightly regulated by central energy/metabolism signaling networks [33]. In parallel, drought-responsive protein changes are intimately linked to the dynamics of reactive oxygen and nitrogen species (ROS/RNS), which serve as both signaling molecules and agents of oxidative modification. Under water deficit, enhanced ROS/RNS generation in chloroplasts, mitochondria, and peroxisomes triggers oxidative post-translational modifications (e.g., carbonylation, S-nitrosylation, and sulfenylation) that can alter protein activity, stability, and turnover [34]. The precise balance between ROS/RNS production and antioxidant scavenging systems (including superoxide dismutase, catalase, and ascorbate-glutathione cycle enzymes) determines whether these reactive species act as beneficial signals or cause irreversible damage. Efficient redox homeostasis is therefore essential to protect photosynthetic proteins from over-oxidation while allowing moderate oxidative signals to activate stress-adaptive programs. Similarly, the synchronized down-regulation and substantial recovery of the R-C4/P-C2 pair during the drought and rewatering cycle, encoding core photosynthetic components, reveal the dual nature of the photosynthetic apparatus—both vulnerable to water deficit yet capable of remarkable resilience. This behavior reflects a canonical photoprotective strategy: the photosynthetic machinery is rapidly deactivated under drought to prevent irreversible photodamage, a process that inherently enables its partial functional recovery upon rewatering [35].

A pivotal finding of our study lies in the widespread transcriptome–proteome discordance that unfolds under drought stress. Our temporal analyses revealed substantial discordance between transcriptional and translational responses, while the protein-to-RNA ratio analysis indicated stable translation efficiency across conditions. Further investigation using median absolute deviation (MAD) showed markedly increased dispersion in PTR distribution under drought stress, demonstrating a pronounced decoupling between transcriptional and translational control. Functional characterization revealed that genes with high PTR maintained enrichment in ribosomal functions, protein folding, photosynthesis, and energy metabolism, whereas low-PTR genes were linked to transcriptional regulation and metabolic adjustment, indicating a clear functional division in translational efficiency. The strategic enrichment of discordant genes in photosynthesis and energy metabolism implies a resource-allocation mechanism under drought stress. The coordinated transcriptional down-regulation of photosynthetic components, coupled with PTM-mediated stabilization or suppression of corresponding proteins, suggests a dual strategy of energy conservation and functional readiness. Mechanistically, phosphorylation directly controls protein turnover—for instance, the phosphorylation status of PSII core proteins directly influences their degradation and the progression of the repair cycle [36]. Under energy-limited drought conditions, SnRK1 phosphorylates translation initiation factors eIF4E and eIFiso4E, suppressing cap-dependent translation to conserve resources [37]. Acetylation, in turn, stabilizes proteins via N-terminal masking of degrons, preventing ubiquitin-proteasome recognition and extending half-life [38]. Collectively, these PTM-driven mechanisms directly contribute to the observed decoupling between steady-state transcript levels and functional protein abundance, enabling plants to rapidly adjust protein outputs without altering transcription.

This aligns with established roles of dynamic PTMs in reprogramming signaling and metabolic networks under stress [39,40]. A notable feature of our data is that transcript–protein discordance was far more pronounced for photosynthesis and energy metabolism than for other functional groups. This bias likely stems from several inherent properties of these pathways. Photosynthetic proteins turn over extremely rapidly; for instance, the D1 subunit of photosystem II has a half-life of only 30–60 min under light, requiring continuous translation [41], while drought disrupts organellar translation elongation by oxidizing key elongation factors, creating a temporal mismatch between stable transcripts and labile proteins [42]. Metabolic pathways must adjust within seconds to minutes to avoid toxic intermediate accumulation, a speed at which PTM-based regulation operates efficiently [43]. Furthermore, synthesizing abundant photosynthetic complexes demands enormous energy and nitrogen [44]; under stress, maintaining mRNA reserves while transiently suppressing translation via PTMs—a “functional pausing” strategy—is metabolically advantageous. The endosymbiotic origin of chloroplasts adds further regulatory complexity, as nuclear and organellar genomes must be coordinated [45]. PTMs directly implement these adaptive strategies: phosphorylation of PSII and LHCII components by kinases such as STN7 and STN8 is essential for maintaining photosynthetic excitation balance and facilitating repair of photodamaged proteins under adverse conditions [25]. Concurrently, recent evidence shows that heat stress-induced deacetylation, mediated by HDA714, enhances rice thermotolerance by activating glycolytic flux [46]. The significant correlations we observed between PTM dynamics and residual changes in protein abundance further substantiate their role as post-transcriptional tuners.

A growing body of evidence highlights the prevalence of discordance between RNA and protein levels in plants. In Arabidopsis, for instance, codon usage and mRNA abundance collectively explain approximately 50% of the variation in protein levels, leaving a substantial proportion under post-transcriptional control [47]. Further complexity is illustrated in rice, where m^6^A methylation in untranslated regions correlates negatively with protein abundance, contributing to RNA–protein divergence [16]. Similarly, integrative analyses in soybean have uncovered novel m^6^A-mediated regulatory modules [17]. Importantly, in the context of abiotic stress, a comprehensive study in rice revealed that salt stress induces dynamic changes in RNA modifications (m^6^A and m^5^C), which in turn significantly enhance the translation efficiency of modified mRNAs, establishing a direct link between the epitranscriptome and protein output under stress conditions [48]. Our work extends this paradigm by demonstrating that phosphorylation and acetylation are specifically enlisted to buffer protein output for critical functional categories, enabling precise tuning of gene expression in fluctuating environments.

## 4. Materials and Methods

### 4.1. Plant Materials and Drought–Rewatering Cycle Treatment

Fully filled and uniform rice seeds (*Oryza sativa* L. cv. Nipponbare) were surface-sterilized with 0.05% (*v*/*v*) NaClO for 10 min and soaked in water for 3 days until germination. Uniform seedlings were planted in trays containing a mixture of vermiculite and peat soil (1:1, *v*/*v*) and grown in a controlled growth chamber at 30 °C (day)/26 °C (night) under a long-day photoperiod (16 h light/8 h dark) with a light intensity of 200 μmol·m^−2^·s^−1^ (white LED) measured at canopy height. When the first tiller emerged, seedlings were transferred to soil-filled cultivation buckets filled with commercial rice soil (organic matter ≥ 30%, pH 5.8–7.0) and grown with regular fertilization until the panicle initiation stage. All plants were grown under the same controlled conditions (30 °C/26 °C, 16 h light/8 h dark, 500 μmol·m^−2^·s^−1^) throughout the experiment. To impose drought stress, irrigation was withheld until approximately 80% of the leaves per plant showed visible wilting (leaf drooping and rolling) and leaf water potential declined to stress levels as measured by a WP4C dew point water potential meter (METER, Pullman, WA, USA), serving as the primary biological endpoint criteria for consistent drought severity across biological replicates. Representative photographs of control, drought-stressed, and rewatered plants are provided in Figure S8. Rewatering was then applied, and these plants were designated as the drought-recovery group (DTR). One-third of the seedlings were continuously maintained under well-watered conditions as the control group (CK). For all three treatments, the top three fully expanded leaves were used for physiological measurements, and leaves from each set of five seedlings were pooled as one biological replicate for subsequent analyses.

### 4.2. Measurement of Gas Exchange Parameters and Water Potential

Gas exchange parameters, including the net photosynthetic rate (Pn) and stomatal conductance (Gs) of the leaves were measured using a portable photosynthesis system (LI-6800, Li-COR, Lincoln, NE, USA). Light intensities were set at 1500 μmol/(m^2^·s), the temperature in the chamber was controlled at 28 °C, and the CO_2_ concentration was set as 400 μmol/mol [49]. For each treatment group, three leaves with consistent growth were selected. Each leaf was measured three to four times, and the results were averaged to obtain reliable gas exchange data. The water potential measurement was obtained using a WP4C dew point water potential meter (METER, Pullman, WA, USA). For each treatment group, two or three leaves with consistent growth were selected and measured following the manufacturer’s instructions. All data were calculated and analyzed using a one-way ANOVA test (analysis of variance) with Prism9 software (ver. 9.4.1).

### 4.3. Transcriptome Sequencing (RNA-Seq) Library Construction

Total RNA was isolated using the *TransZol* Up Plus RNA Kit according to the manufacturer’s instructions (ER501, Transgen, Beijing, China). Subsequently, 1 μg total RNA was used for library construction following the manufacturer’s instructions of VAHTS Universal V8 RNA-seq Library Prep Kit for Illumina (NR605, Vazyme, Nanjing, China), following the manufacturer’s instructions with incorporation of dUTP during second-strand cDNA synthesis to preserve strand information. The resulting libraries were quantified, pooled, and sequenced on an Illumina HiSeq X-Ten platform with a 150 bp paired-end strategy (sequencing service was provided by OE Biotech Co., Ltd., Shanghai, China).

### 4.4. RNA-Seq Data Processing

Raw sequencing reads were quality-checked and trimmed using Trim Galore (v0.6.7) with the parameters “-q 30 --clip_R1 15 --clip_R2 15” to remove adaptor sequences and low-quality bases. Clean reads were then aligned to the *Oryza sativa* reference genome (Os-Nipponbare-Reference-IRGSP-1.0) using HISAT2 (v2.2.1) [50] with default settings and the parameter “--rna-strandness RF” to retain strand-specific information. Transcripts were assembled and count using featureCounts [51] in UGA (formerly MSU) Rice Genome Annotation Release 7 and IRGSP 1.0 (RAP) annotation. The read counts of the genes were then submitted to R package DEseq2 [52] to determine the differentially expressed genes (DEGs) among the treatments based on its negative binomial model with a threshold of |log_2_(fold change)| > 1.5 and *p*-adjust (Wald test *p*-value adjusted by Benjamini–Hochberg method) < 0.05. See Supplementary Tables S1 and S2 for RNA-seq sample summary for two genome reference.

### 4.5. Protein Extraction and Peptides Preparation

Frozen leaf samples were ground thoroughly in liquid nitrogen to fine powder and transferred into 1.5–2 mL microcentrifuge tubes. Each sample was mixed with 800 µL phenol extraction buffer supplemented with phosphatase inhibitors and 1 mM PMSF, followed by the addition of an equal volume of Tris–phenol (pH 7.8). The mixtures were incubated at 4 °C for 40 min with intermittent vortexing and centrifuged at 7100 rpm for 10 min at 4 °C. The upper phenol phase was collected and proteins were precipitated overnight at −40 °C with five volumes of 0.1 M ammonium acetate in methanol. After 12,000 rpm centrifugation for 10 min at 4 °C, the pellets were washed once with cold methanol and twice with cold acetone to remove contaminants. The air-dried pellets were dissolved in lysis buffer containing 1 mM PMSF, centrifuged twice, and the supernatants were collected as total protein extracts and stored at −80 °C. Protein concentrations were determined using the BCA assay according to the manufacturer’s protocol. Equal protein amounts from each sample were adjusted to the same concentration, reduced with 5 mM DTT at 55 °C for 30 min, and alkylated with 10 mM iodoacetamide in the dark for 15 min at room temperature. Proteins were then precipitated with six volumes of pre-chilled acetone and incubated at −20 °C overnight. The precipitated proteins were collected by centrifugation at 8000× *g* for 10 min at 4 °C, air-dried, and redissolved in 50 mM ammonium bicarbonate. Trypsin was added at a 1:50 (enzyme:protein, *w*/*w*) ratio, and digestion was performed at 37 °C overnight. The reaction was stopped by adjusting the pH to ~3 with phosphoric acid.

### 4.6. Peptide Desalting, LC–MS/MS Analysis, and Data Processing

After tryptic digestion, peptides were desalted using a SOLA™ SPE 96-well plate (60309, Thermo Fisher Scientific, Waltham, MA, USA). Columns were activated with 200 μL methanol three times, equilibrated with 200 μL of 0.1% formic acid in water three times, and samples (50–500 μL) were loaded twice at a controlled vacuum (flow rate ~1 mL/min). The columns were washed with 200 μL of 0.1% formic acid in water three times and eluted with 150 μL of 50% acetonitrile containing 0.1% formic acid, repeated three times. Eluates (~450 μL total) were vacuum-dried before LC–MS/MS analysis.

Prior to mass spectrometry, iRT calibration peptides (Biognosys, Newton, MA, USA) were spiked into each sample at a ratio of 1:20 (*v*/*v*) for chromatographic calibration and quantitative quality control. LC separation was performed on a C18 analytical column (15 cm × 100 μm, 1.9 μm) using an EASY-nLC system with buffer A (0.1% formic acid in water) and buffer B (0.1% formic acid in acetonitrile). Peptides were eluted with the following gradient: 0–7.5 min, 6–28% B; 7.5–8.0 min, 28–80% B; 8.0–9.0 min, 80% B; and 9.1–10.0 min, 2% B, at a constant flow rate of 0.7 μL/min.

Separated peptides were analyzed on a timsTOF HT mass spectrometer (Bruker, Berlin, Germany) in data-independent acquisition (DIA) mode. The capillary voltage was set to 1.6 kV, dry gas flow to 3.2 L/min, and dry temperature to 180 °C. MS scans were acquired over a mass range of 300–1500 *m*/*z* with an ion mobility range of 0.7–1.3, collision energy ramped between 20 and 59 eV, and a ramp time of 50 ms.

Raw DIA data were processed using DIA-NN software (version 1.8.1). Spectral libraries were searched against the *Oryza sativa* subsp. *Japonica* UniProt proteome (UP000059680, 2024_11_12.fasta). Trypsin was specified as the digestion enzyme, allowing one missed cleavage. Carbamidomethylation of cysteine was set as a fixed modification, and oxidation (M) and N-terminal acetylation were defined as variable modifications. A target-decoy strategy was used for database searching, with peptide-spectrum match (PSM) and protein-level false discovery rates (FDRs) controlled at 1%.

### 4.7. Phosphopeptide Enrichment and LC–MS/MS Analysis

Digested peptides were first desalted on Sep-Pak C18 cartridges: columns were activated with 600 µL methanol (×2), equilibrated with 600 µL 50% acetonitrile/0.1% formic acid (×2) and 600 µL 0.1% formic acid in water (×2). Samples were loaded by gravity twice, washed with 600 µL 0.1% formic acid in water (×3) and eluted by gravity with 2 × 600 µL 50% acetonitrile/0.1% formic acid; eluates were vacuum-dried and stored at −80 °C. Lyophilized peptides were resuspended in 200 µL Binding/Wash buffer (80% acetonitrile, 5% trifluoroacetic acid) and phosphopeptides were enriched using High-Select Fe-NTA Phosphopeptide Enrichment Kit (A32992, Thermo Fisher Scientific, Waltham, MA, USA) spin affinity columns: columns were cleared of storage buffer (1000× *g*, 30 s) and equilibrated twice with 200 µL Binding/Wash buffer (1000× *g*, 30 s). Peptide suspension (200 µL) was added, the resin was gently mixed by tapping and incubated 30 min with gentle mixing every 10 min, and then columns were centrifuged (1000× *g*, 30 s). Columns were washed 3× with 200 µL Binding/Wash buffer and once with 200 µL LC-MS water, and phosphopeptides were eluted with 2 × 100 µL Elution buffer, eluates were immediately dried in a vacuum concentrator. iRT peptides (Biognosys, Newton, MA, USA) were spiked at 1:20 (*v*/*v*) before LC. Peptides were separated on a C18 column (15 cm × 100 µm, 1.9 µm) using 0.1% FA in water (A) and 0.1% FA in ACN (B) with the following gradient: 0.0 min, 4% B (0.7 µL/min); to 26.5 min, 28% B (flow 0.4–0.7 µL/min); 26.5–28.0 min to 80% B; 28.0–29.0 min, 80% B; and re-equilibrate to 2% B by 29.5–30.0 min (0.7 µL/min). DIA acquisition was performed on a timsTOF HT (Bruker, Berlin, Germany) with capillary 1.75 kV, dry temp 180 °C, dry gas 3 L/min, mass range 100–1700 *m*/*z*, ion mobility 0.6–1.6, CE 20–60 eV, and ramp time 100 ms. Raw DIA data were processed in Spectronaut Pulsar (v18.7, Biognosys, Newton, MA, USA) against the *Oryza sativa* subsp. *Japonica* UniProt proteome (UP000059680, 2024_11_12.fasta) using Trypsin/P, max missed cleavages = 2, fixed carbamidomethyl (C), variable oxidation (M) and acetyl (protein N-term), phospho (S, T, Y) and epsilon-acetylation (K) as additional variable modifications, precursor and protein Q-value cutoffs = 0.01, and MS2-level quantification.

### 4.8. Acetylpeptide Enrichment and LC–MS/MS

Tryptic peptides were desalted on Sep-Pak C18 cartridges prior to enrichment: cartridges were activated with 600 µL methanol (×2), equilibrated with 600 µL 50% acetonitrile/0.1% formic acid (×2) and 600 µL 0.1% formic acid in water (×2). Samples were loaded by gravity twice, washed with 600 µL 0.1% formic acid in water (×3) and eluted by gravity with 2 × 600 µL 50% acetonitrile/0.1% formic acid; eluates were vacuum-dried and stored at −80 °C. For acetyl-lysine enrichment, reagents from the PTMScan^®^ Acetyl-Lysine Motif (Ac-K) kit (13416, Cell Signaling Technology, Danvers, MA, USA) were thawed at 4 °C. The 10× IAP buffer was diluted to 1× with prechilled water and antibody immunoaffinity beads were washed with cold PBS to remove glycerol. Lyophilized peptides were dissolved in 1.4 mL 1× IAP buffer and incubated with the antibody beads at 4 °C with end-over-end rotation for 2 h. After centrifugation (2000× *g*, 30 s), the supernatant was removed and stored at −80 °C; beads were washed twice with 1× IAP buffer and three times with cold water (all washes at 4 °C). Acetylated peptides were eluted twice with 40 µL 0.15% TFA (2 × 40 µL), centrifuged (2000× *g*, 30 s), desalted using Pierce™ C18 Spin Tips, and vacuum dried. Prior to LC–MS/MS, iRT peptides (Biognosys, Newton, MA, USA) were spiked into each sample at a 1:20 (*v*/*v*) ratio for retention-time calibration. Peptides were separated on a C18 analytical column (15 cm × 100 µm, 1.9 µm) using buffer A (0.1% FA in water) and buffer B (0.1% FA in acetonitrile) with the gradient: 0.0 min, 6% B; to 27.5 min, 28% B; 29.0 min, 80% B; and hold to 30.0 min, 80% B (flow rate 0.7 µL/min). DIA acquisition was performed on a timsTOF HT (Bruker, Berlin, Germany) with capillary voltage 1.75 kV, dry temperature 180 °C, dry gas 3 L/min, mass range 100–1700 *m*/*z*, ion mobility 0.6–1.6, collision energy ramp 20–60 eV and ramp time 100 ms. Raw DIA files were processed in Spectronaut Pulsar (v18.7, Biognosys, Newton, MA, USA) against the *Oryza sativa* subsp. *Japonica* UniProt proteome (UP000059680; 2024_11_12.fasta) using Trypsin/P (max missed cleavages = 2), fixed modification carbamidomethyl (C), variable modifications oxidation (M) and protein N-terminal acetylation, with precursor and protein Q-value cutoffs of 0.01 and MS2-level quantification.

### 4.9. Proteome Data Processing

Protein quantification data were preprocessed by filtering proteins or sites with at least two valid values across all samples and valid values ≥50% in at least one group. Missing values were imputed using group means when valid values were ≥50%, and the remaining missing values were replaced with half of the global minimum intensity. The data were then median-normalized, log_2_-transformed, and shifted by adding 10 plus the absolute value of the minimum log_2_ intensity (*log*_2_(*x*) + 10 + |*min*(*log*_2_(*x*))|) to ensure all values were positive and comparable across samples. See Supplementary Table S3 for proteome data analysis and Supplementary Tables S4 and S5 for phosphoproteome and acetylome data analysis.

### 4.10. Statistical Analysis

All experimental data were visualized using Prism 9 and analysis of variance (two-way ANOVA) was performed using IBM SPSS Statistics 26.0. A difference of *p*  <  0.05 was considered significant. Each of the above experimental results is calculated from two or three replicates.

## Data Availability

The raw data that support the findings of this study have been deposited in the Genome Sequence Archive in National Genomics Data Center, China National Center for Bioinformation/Beijing Institute of Genomics, Chinese Academy of Sciences (GSA: CRA042099 and OMIX: OMIX016543, OMIX016544, OMIX016545) that are publicly accessible at https://ngdc.cncb.ac.cn/gsa.

## Supplementary Materials

The following supporting information can be downloaded at https://www.mdpi.com/article/10.3390/plants15101559/s1: Figure S1: The quality control of RNA-seq libraries data; Figure S2: The characterization of Pro-DIA data; Figure S3: Quantitative comparison of RNA expression and protein abundance during drought and watering cycle; Figure S4: The characterization of PTR and GO enrichment across PTR levels; Figure S5: The characterization of phosphoproteome data; Figure S6: The characterization of acetylome data; Figure S7: Comparison of the numbers of canonical transcription factors identified at the RNA, protein, phosphoproteome and acetylome levels across treatments; Figure S8: Representative morphology and leaf water potential of rice plants under control, drought, and rewatering conditions; Table S1: Summary of RNA-seq expression levels (TPM) counted in Rice Genome Annotation Release 7 (MSU7) for all detected transcripts under control, drought stress, and rewatering treatments; Table S2: Summary of RNA-seq expression levels (TPM) counted in IRGSP-1.0 (RAP) annotation for all detected transcripts under control, drought stress, and rewatering treatments; Table S3: Summary of Pro-DIA abundance data for all detected peptides or proteins under control, drought stress, and rewatering treatments; Table S4: Summary of phosphoproteome abundance data for all detected peptides or proteins under control, drought stress, and rewatering treatments; Table S5: Summary of acetylome abundance data for all detected peptides or proteins under control, drought stress, and rewatering treatments.
