# Supplementary material for: Proteome–Transcriptome Discordance in Rice Under Drought Is Modulated by Post-Translational Modifications with Functional Consequences for Photosynthesis and Energy Metabolism
Source: Plants (Basel). 2026 May 20;15(10):1559. doi: 10.3390/plants15101559 (PMC13210473; doi:10.3390/plants15101559)
Supplement: Supplementary file 1 [file plants-15-01559-s001.zip › Supplementary Figures.pdf]

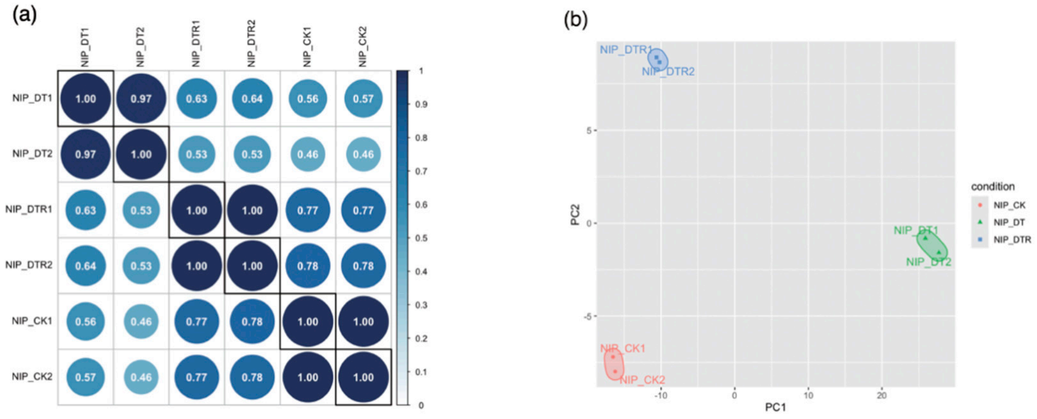

Figure S1. The quality control of RNA-seq libraries' data. (a) Pearson correlation matrix of RNA-seq data across treatments. (b) Principal component analysis (PCA) of RNA-seq samples across treatments.

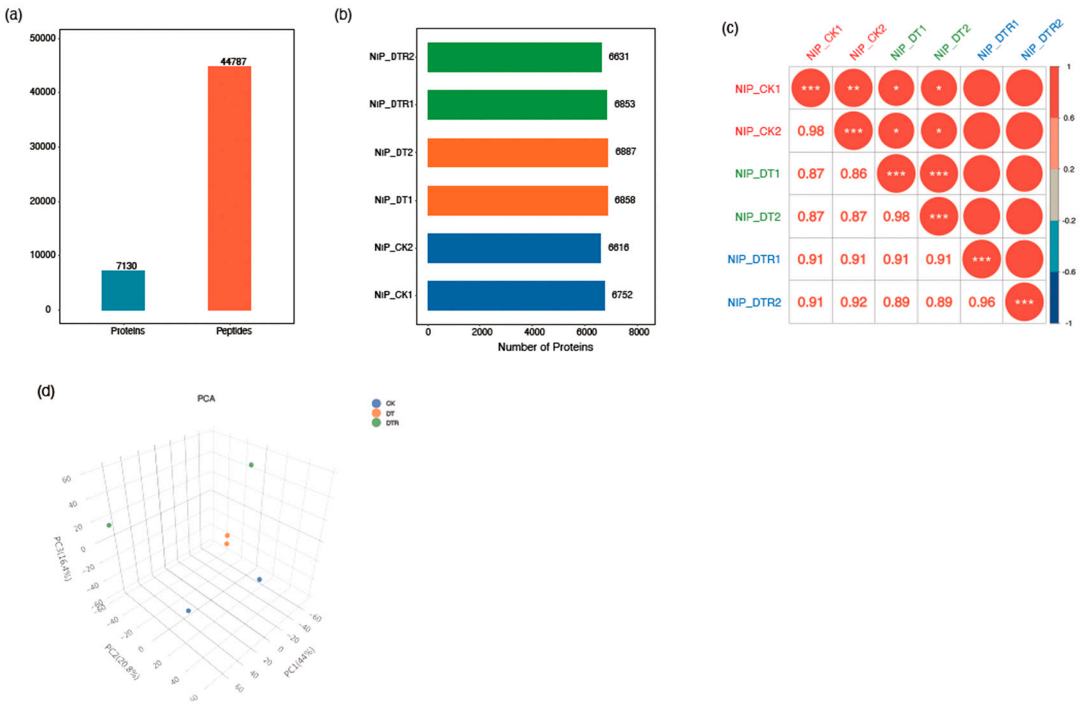

Figure S2. The characterization of Pro-DIA data. (a) The total numbers of peptides and proteins identified by Pro-DIA. (b) The numbers of proteins identified by Pro-DIA in each replicate across treatments. (c) Pearson correlation matrix of Pro-DIA data across treatments. (d) Principal component analysis (PCA) of Pro-DIA samples across treatments.

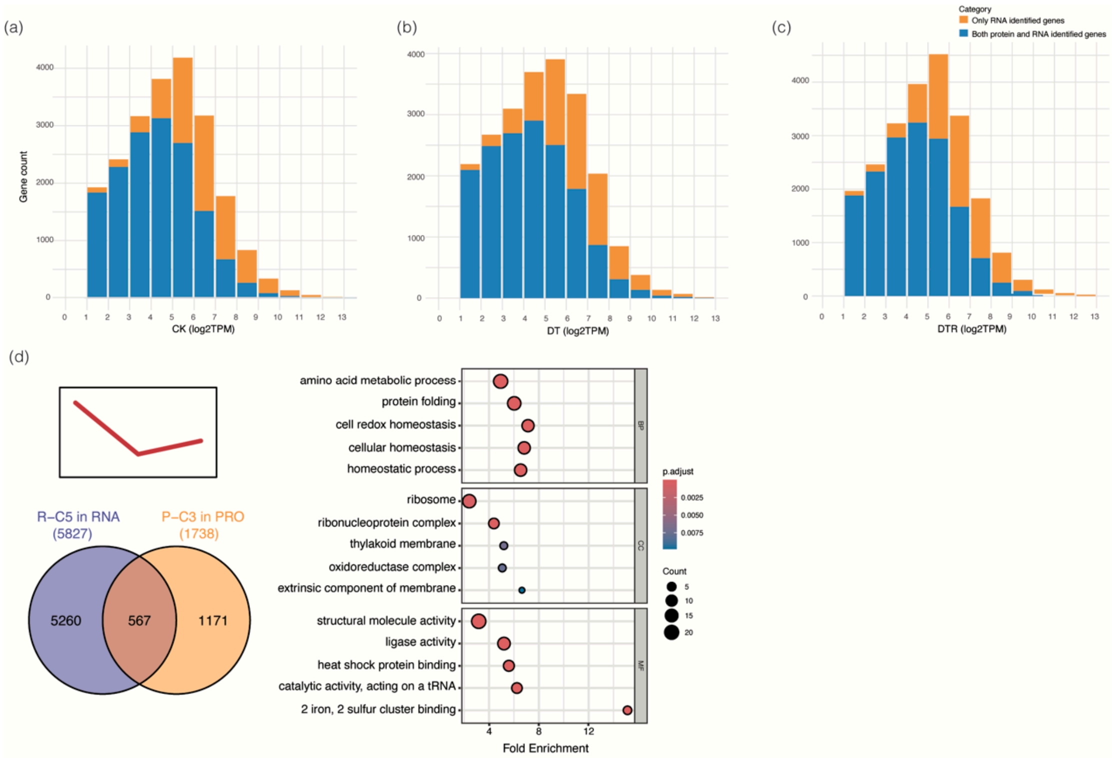

Figure S3. Quantitative comparison of RNA expression and protein abundance during drought and watering cycle. (a) A comparison of gene number between genes with both protein and RNA expression and genes with only RNA expression in CK. The RNA expression levels were transformed using $\log_2$ (TPM + 1) and the RNAs with $\log_2$ (TPM + 1) <1 were adjusted to 1. (b) A comparison of gene number between genes with both protein and RNA expression and genes with only RNA expression in DT. The RNA expression levels were transformed using $\log_2$ (TPM + 1) and the RNAs with $\log_2$ (TPM + 1) <1 were adjusted to 1. (c) A comparison of gene number between genes with both protein and RNA expression and genes with only RNA expression in DTR. The RNA expression levels were transformed using $\log_2$ (TPM + 1) and the RNAs with $\log_2$ (TPM + 1) <1 were adjusted to 1. (d) The Venn diagram shows the overlap of genes whose RNA and protein expression were both down-regulated under drought and exhibited only partial recovery after rewatering, corresponding to the R-C5/P-C3 cluster pair and GO enrichment analysis of the shared genes and proteins in this pattern.

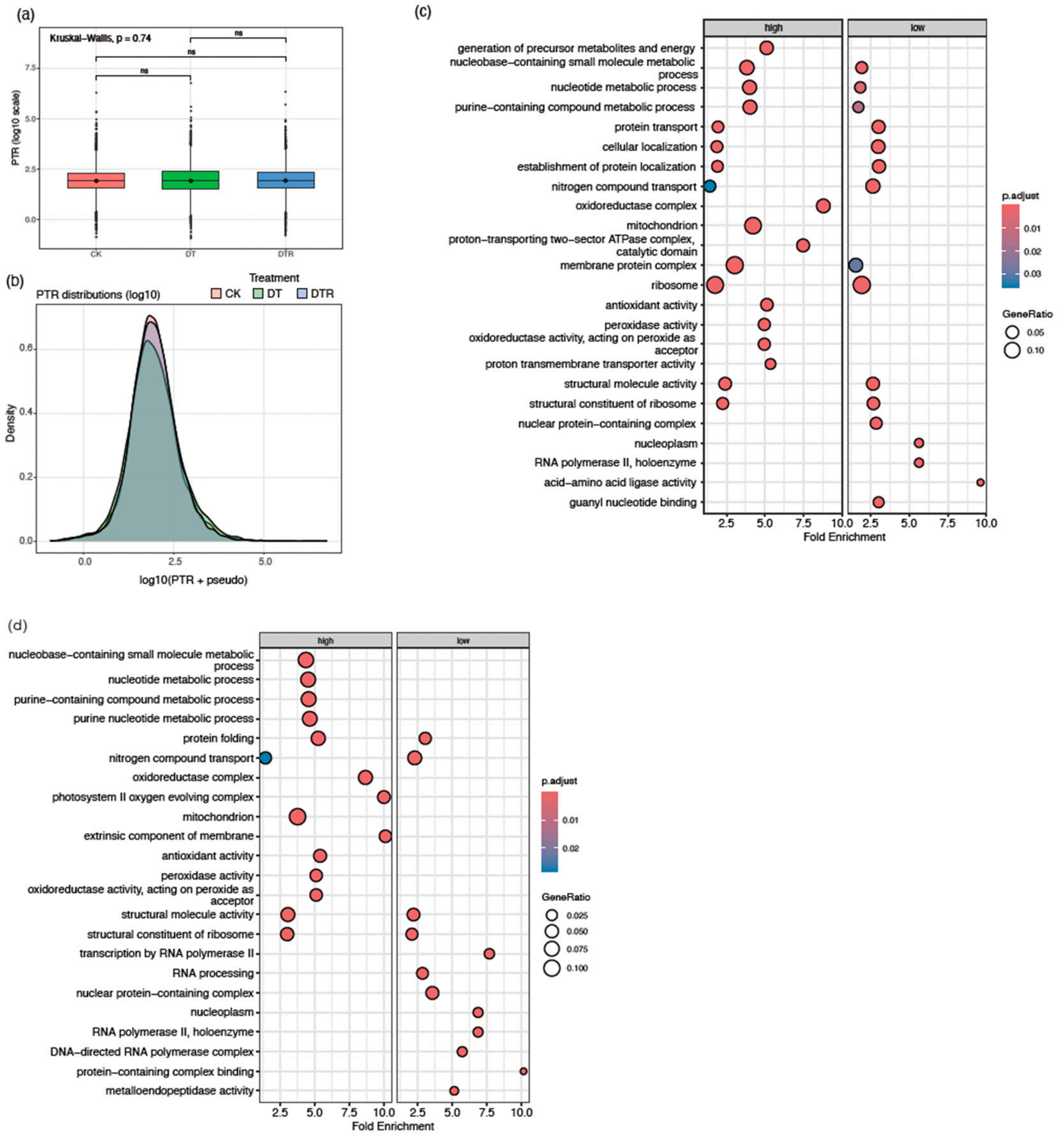

Figure S4. The characterization of PTR and GO enrichment across PTR levels. (a) The PTR variation across treatments. (b) The PTR distribution across treatments. (c) GO enrichment analysis of high-PTR and low-PTR genes under control treatment. (d) GO enrichment analysis of high-PTR and low-PTR genes under rewatering treatment.

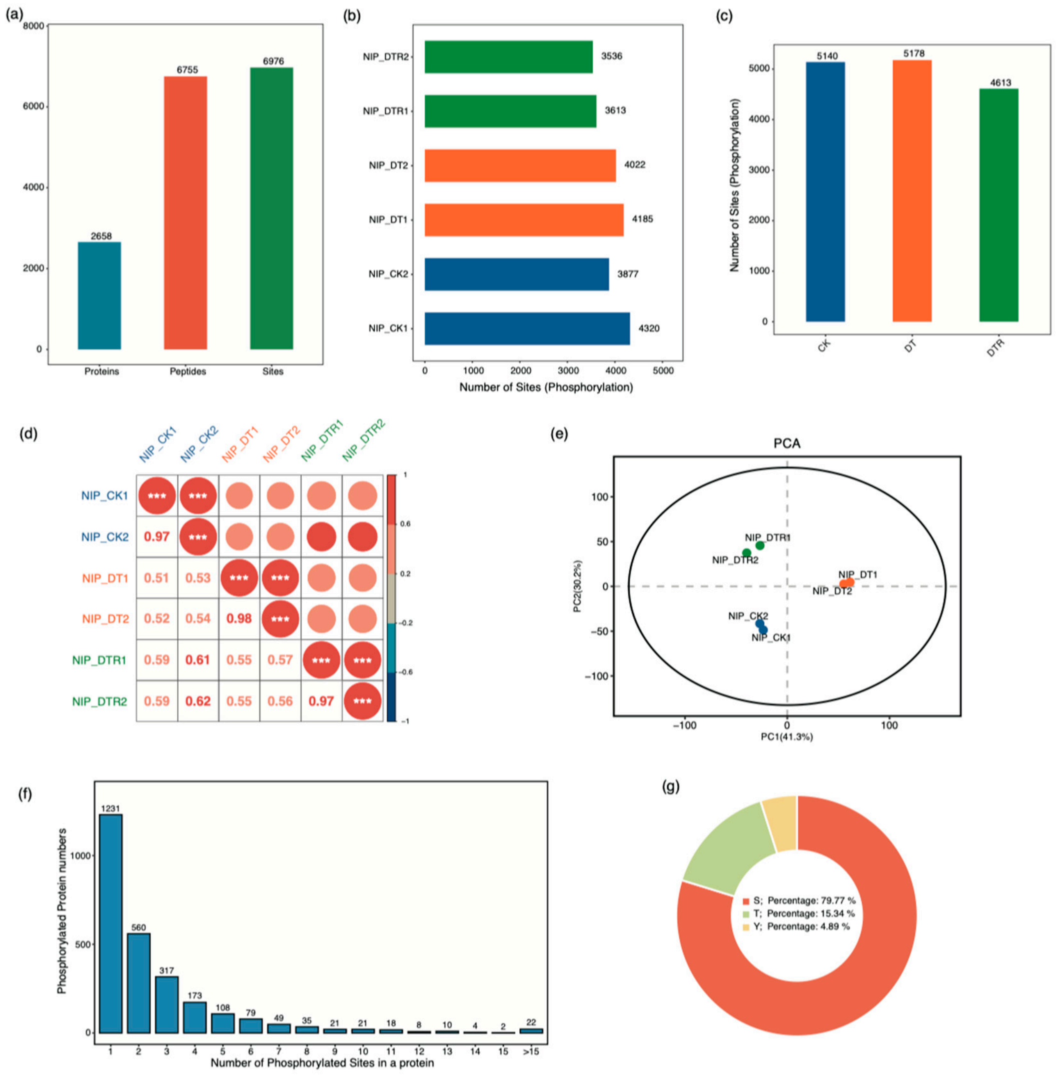

Figure S5. The characterization of phosphoproteome data. (a) The total numbers of phosphorylated peptides, phosphorylated proteins and phosphorylated sites identified by phosphoproteome. (b) The numbers of phosphorylated sites identified by phosphoproteome in each replicate across treatments. (c) The total numbers of phosphorylated sites identified by phosphoproteome across treatments. (d) Pearson correlation matrix of phosphoproteome data across treatments. (e) Principal component analysis (PCA) of phosphoproteome samples across treatments. (f) The distribution of the number of phosphorylation sites per protein. (g) The phosphorylation modification ratio in different amino acids.

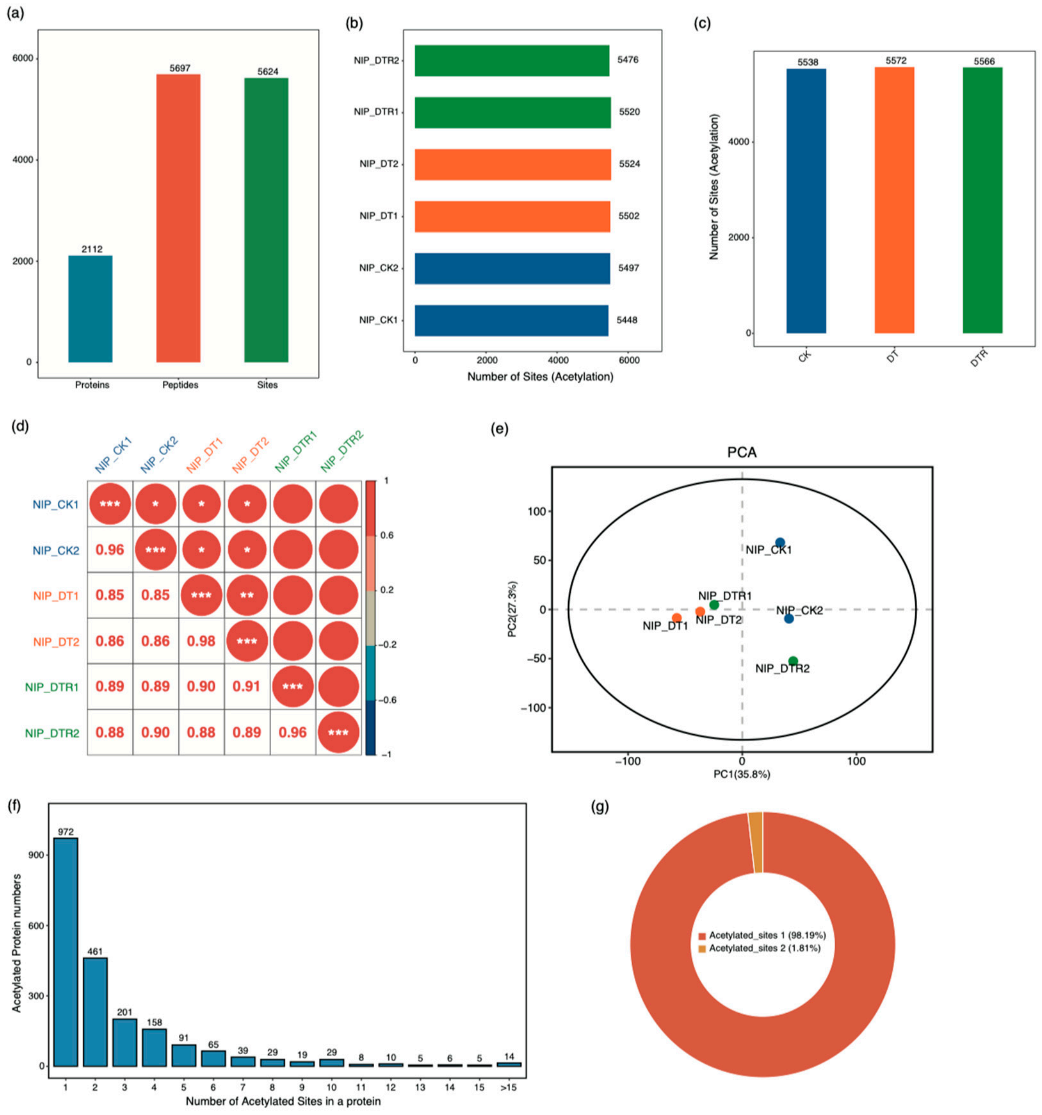

Figure S6. The characterization of acetylome data. (a) The total numbers of acetylated peptides, acetylated proteins and acetylated sites identified by acetylome. (b) The numbers of acetylated sites identified by acetylome in each replicate across treatments. (c) The total numbers of acetylated sites identified by acetylome across treatments. (d) Pearson correlation matrix of acetylome data across treatments. (e) Principal component analysis (PCA) of acetylome samples across treatments. (f) The distribution of the number of acetylation sites per protein. (g) The number of acetylation sites per protein.

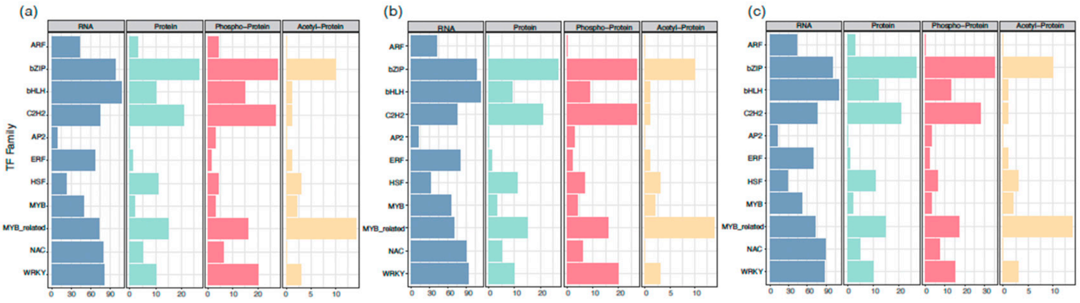

Figure S7. Comparison of the numbers of canonical transcription factors identified at the RNA, protein, phosphoproteome and acetylome levels across treatments. (a-c) Comparison of the numbers of canonical transcription factors identified at the RNA, protein, phosphoproteome and acetylome levels across CK (a), DT (b) and DTR.
