# Supplementary figures and images for: Proteome–Transcriptome Discordance in Rice Under Drought Is Modulated by Post-Translational Modifications with Functional Consequences for Photosynthesis and Energy Metabolism
Source: Plants (Basel). 2026 May 20;15(10):1559. doi: 10.3390/plants15101559 (PMC13210473; doi:10.3390/plants15101559)

(a)

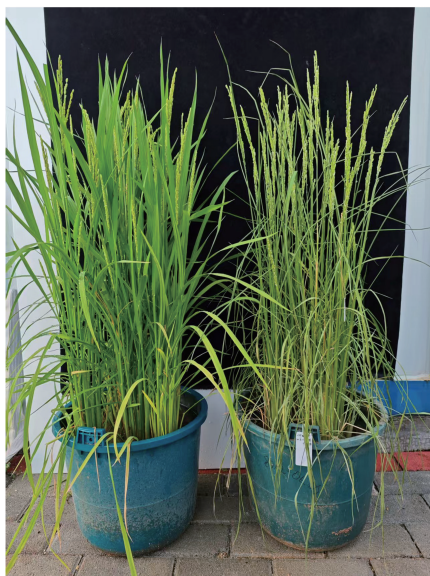

CK

DT

(b)

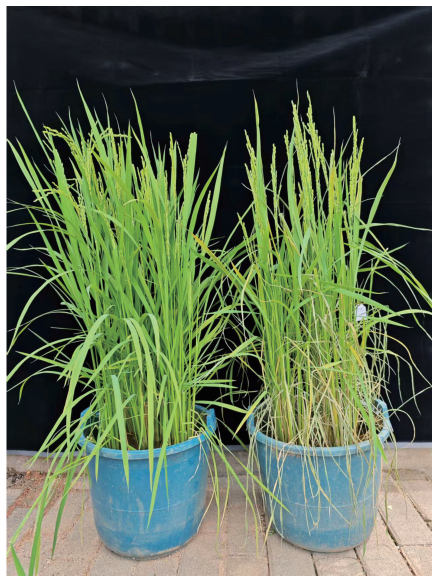

CK

DTR

(c)

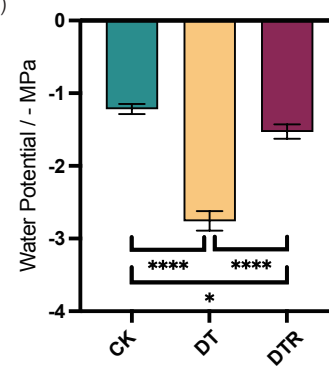

Supplement: Supplementary file 1 [file plants-15-01559-s001.zip › FigureS8.pdf]
